# Inhibition of HCV Replication by Oxysterol-Binding Protein-Related Protein 4 (ORP4) through Interaction with HCV NS5B and Alteration of Lipid Droplet Formation

**DOI:** 10.1371/journal.pone.0075648

**Published:** 2013-09-17

**Authors:** In-Woo Park, Jean Ndjomou, Yahong Wen, Ziqing Liu, Neale D. Ridgway, C. Cheng Kao, Johnny J. He

**Affiliations:** 1 Department of Microbiology and Immunology, Indiana University School of Medicine, Indianapolis, Indiana, United States of America; 2 Center for AIDS Research, Indiana University School of Medicine, Indianapolis, Indiana, United States of America; 3 University of North Texas Health Science Center, Fort Worth, Texas, United States of America; 4 Department of Molecular and Cellular Biochemistry, Indiana University, Bloomington, Indiana, United States of America; 5 Department of Biochemistry and Molecular Biology, Dalhousie University, Halifax, Nova Scotia, Canada; Istituto Nazionale per le Malattie Infettive, Italy

## Abstract

Hepatitis C virus (HCV) RNA replication involves complex interactions among the 3’x RNA element within the HCV 3’ untranslated region, viral and host proteins. However, many of the host proteins remain unknown. In this study, we devised an RNA affinity chromatography /2D/MASS proteomics strategy and identified nine putative 3’ X-associated host proteins; among them is oxysterol-binding protein-related protein 4 (ORP4), a cytoplasmic receptor for oxysterols. We determined the relationship between ORP4 expression and HCV replication. A very low level of constitutive ORP4 expression was detected in hepatocytes. Ectopically expressed ORP4 was detected in the endoplasmic reticulum and inhibited luciferase reporter gene expression in HCV subgenomic replicon cells and HCV core expression in JFH-1-infected cells. Expression of ORP4S, an ORP4 variant that lacked the N-terminal pleckstrin-homology domain but contained the C-terminal oxysterol-binding domain also inhibited HCV replication, pointing to an important role of the oxysterol-binding domain in ORP4-mediated inhibition of HCV replication. ORP4 was found to associate with HCV NS5B and its expression led to inhibition of the NS5B activity. ORP4 expression had little effect on intracellular lipid synthesis and secretion, but it induced lipid droplet formation in the context of HCV replication. Taken together, these results demonstrate that ORP4 is a negative regulator of HCV replication, likely via interaction with HCV NS5B in the replication complex and regulation of intracellular lipid homeostasis. This work supports the important role of lipids and their metabolism in HCV replication and pathogenesis.

## Introduction

Hepatitis C virus (HCV) causes chronic liver diseases such as steatosis, cirrhosis, and hepatocellular carcinoma [1,2]; 170 million people worldwide are currently infected with the virus (1). HCV is a (+) single-stranded RNA virus that belongs to the *hepacivirus* genus of the *Flaviviridae* family [3]. The viral genome is approximately 9.6 Kb. The 5' untranslated region (UTR) contains an internal ribosome entry site (IRES), which allows internal initiation of translation to express a 3010 amino acid polyprotein, while the 3’ UTR has a tripartite structure composed of a variable region, a poly-U/UC tract, and a highly conserved 3’ X region of 98 nucleotides in length that is essential for RNA replication [4,5]. The polyprotein is then processed co- and post-translationally by a combination of viral and host proteases into three structural proteins Core, E1 and E2, and seven nonstructural (NS) proteins p7, NS2, NS3, NS4A, NS4B, NS5A, and NS5B [6,7]. Each of these HCV proteins serves distinct functions in multiple steps of the HCV life cycle such as entry, uncoating, translation, replication, assembly and release.

Evidence has accumulated showing that synthesis of the intermediate RNA strand of negative polarity is regulated by interaction between host cellular factors and the HCV 3’ X region. First, the 3’ X is highly conserved among all the HCV genotypes and forms a stable secondary structure, indicating its important biological function. The conserved 3’ termini have been proposed to be involved in viral RNA replication and in multiple protein-RNA interactions in other positive-stranded RNA viruses [8-11]. Second, mutagenesis analysis of infectious cDNA clones of HCV showed that the 3’ X was indispensable for virus multiplication in the chimpanzee [12,13]. Third, the replication of HCV RNA is believed to occur via its transcription into a complementary, genomic-length RNA, the so-called negative-strand HCV RNA [14].

Similar to other members of the *Flaviviridae* family, a membrane-associated replicase complex initiates replication at the 3’ end of the HCV to synthesize a complementary negative-strand RNA. Two viral nonstructural proteins, NS5B (RNA-dependent RNA polymerase) and NS3 (protease and helicase/NTPase), are the major components of the replicase complex [15-17]. Studies have identified several 3’ X-binding cellular proteins [18], including the La protein [19,20], glyceraldehydes-3-phosphate dehydrogenase [21], hnRNP C [22], polypyrimidine tract-binding protein [23] and human ribosomal proteins [24]. Most of these factors are isolated by an *in vitro* UV-crosslinking assay and are also ubiquitously expressed. Therefore, the exact roles of these factors in HCV replication remain to be determined.

In the present report, we used a novel RNA affinity chromatography strategy in combination with 2D/MASS to identify 3’ X-associated proteins in hepatocytes. Among those isolated from the 3’ X-containing complex was the oxysterol binding protein-related protein 4 (ORP4). Further characterization of the roles of ORP4 in HCV replication revealed that it functions as a negative regulator of HCV replication, likely affecting intracellular lipid re-distribution and homeostasis. This study adds to the growing evidence that lipids play a direct role in the intracellular processes of HCV replication. Moreover, these findings suggest that both the absolute level and the dynamics of the lipids within the cells are important for HCV replication.

## Materials and Methods

### Plasmids

For pCMV-flag-ORP4 and pCMV-flag-ORP4S, plasmid pcDNA3-ORP4 [25] was used as a template for PCR cloning with primers containing a HindIII site and an EcoRV site. The PCR product was digested with Hind III/EcoR V and cloned into corresponding sites of pCMV2-flag vector (Sigma, St Louis, MO). All recombinant plasmids were confirmed by sequencing.

### Cells and Reagents

Clone B, a Huh 7 derived cell line that contains the stably replicating HCV subgenomic replicon was obtained from American Type Culture Collection (ATCC) and propagated in Dulbecco modified Eagle medium (DMEM) supplemented with 10% heat-inactivated fetal bovine serum (FBS), 1 mM sodium pyruvate, 1% penicillin/streptomycin, 1% L-glutamine, 1 mM non-essential amino-acids, 250 µg/ml G418, in a 37°C and 5% CO2 incubator. HCV RLuc subgenomic replicon cells (RLuc) were obtained from Dr. Seng-Lai Tan [26] and were grown in the above conditions. 293T, Huh7.5, and Huh7.5.1 cells were maintained in these same conditions, except for the absence of G418 in the growth medium. Cerulenin (CRLN), methy-β-cyclodextrin (MβCD), and 25-hydroxycholesterol (25-HC; Sigma) were dissolved in chloroform, phosphate-buffered saline (PBS), and chloroform, respectively, according to the manufacturer’s instructions, and were used at the indicated concentrations.

### Preparation of cellular protein extracts for RNA affinity chromatography

Cells were harvested and washed twice in cold PBS. Cell extracts were prepared essentially as described elsewhere [27]. Briefly, cells were lysed in hypotonic buffer (10 mM Tris.HCl pH 7.4, 10 mM NaCl) containing 0.5% NP-40, 1 mM DTT, 1 mM PMSF, and 1x protease inhibitor cocktail (Roche, Indianapolis, IN) on ice for 20 min followed by 5 freeze/thaw cycles. The lysates were centrifuged at 1500*g* at 4°C for 10 min, the supernatant was collected into new tubes, and the pellets were further extracted with a buffer containing 20 mM Tris.HCl pH 7.4, 450 mM NaCl, 1 mM EDTA, 1 mM DTT, 0.5% NP-40, 1 mM PMSF, and 1 x protease inhibitor cocktail on ice for 20 min, and then centrifuged at 10,000*g* at 4°C for 10 min. The supernatants were combined, and the protein concentration determined with the Bio-Rad protein assay kit (Bio-Rad, Hercules, CA) for RNA affinity chromatography.

### Preparation of RNA transcripts and pull-down of 3’X RNA-containing HCV replication complex

The biotinylated 98-nucleotide 3’X RNA was synthesized with the MaxiScript RNA transcription kit (Ambion, Austin, TX) using BamHI-linearized pGEM-3Z-HCV-3’X as the template and the T7 RNA polymerase. The run-off RNA transcripts were thereafter treated with 2 U of RNase-free DNase I at 37°C for 30 min to remove the template DNA and purified by acidic phenol/chloroform extraction followed by ethanol precipitation. Precipitated RNA was suspended in RNase-free water, and the concentration and integrity were determined using 5% polyacrylamide-7.5 M urea gel electrophoresis. The biotin-3’X RNA (15 µg) was attached to pre-equilibrated streptavidin coated agarose beads (Pierce, Rockford, IL) in the presence of binding buffer (10 mM Tris.HCl pH7.5, 150 mM KCl, 1.5 mM MgCl_2_, 0.5 mM DTT, 0.05% NP-40 and 1 mM PMSF) by incubating at 4°C for 2 hr on a rocker. Unbound RNA was removed by washing five times with the binding buffer and incubating 10 min each time with rocking. Bound RNA was analyzed, along with the washes, on a 5% polyacrylamide gel to confirm RNA conjugating efficiency. After washing, protein extracts (1.5 mg) and yeast tRNA (30 µg) were applied to the bound RNA-streptavidin complex and incubated at 4°C for 2 hr with rocking. The beads were washed 5 times with binding buffer and the resin-bound proteins were eluted with the elution buffer (10 mM Tris.HCl pH7.5, 10% glycerol, 2% SDS). The eluted proteins were precipitated, and SDS was removed using a protein clean-up kit (BioRad); the proteins were then suspended in a 2D sample buffer for 2D gel electrophoresis, followed by image analysis and MASS identification.

### Reporter gene assay

The HCV RLuc replicon cells were grown in a 24-well plate and transfected with the indicated amount of each plasmid using the Lipofectamine 2000 transfection reagent (Invitrogen, Carlsbad, CA). The total amount of transfected plasmid DNA was equalized with pcDNA3, and pCMV-βGal plasmid was co-transfected to normalize the transfection efficiency. The Luciferase reporter gene assay was performed using the Dual-Glo Luciferase assay kit (Promega) according to the manufacturer’s instructions. Briefly, cells were collected 48 hr post-transfection, washed twice with ice-cold PBS, and lysed in *Renilla* luciferase lysis buffer. The luciferase activity in the lysates was determined using 20 µl clarified cell lysates and 80 µl *Renilla* luciferase assay buffer with a luminometer (Lumat LB9501 Berthold, Freiburg, Germany). For experiments with lipid synthesis inhibitors or lipid sequestering agent, HCV RLuc subgenomic replicon cells were plated in a 24 well plate and treated with the indicated concentration of 25-HC or CRLN in the DMEM containing charcoal-treated FBS (CD-DMEM) or in the serum-free DMEM (SF-DMEM) for 24 hr, followed by 1 hr treatment with the indicated concentration of MβCD in the SF-DMEM. Cells over-expressing ORP4 were plated in a 24-well plate and transfected with ORP4-expressing plasmid, as described above, cultured for 48 hr, and then treated with the indicated concentration of inhibitors, as earlier stated. All data were normalized to equal levels of lysates and transfection efficiencies. All luciferase reporter gene assays were repeated at least three times.

### Transfection and infection

Huh7.5.1 cells were cultured in a 24-well plate and first transfected with pCMV-flag-ORP4 expression plasmid using the Lipofectamine 2000 transfection reagent (Invitrogen). Four hours post transfection, the cells were infected with JFH-1 at an MOI of 0.5 for additional 4 hr. The total amount of plasmid DNA was equalized with pcDNA3, and pc3.GFP plasmid was co-transfected to ensure a comparable transfection efficiency. Cells were then collected for MTT assay or Western blot analyses 3 days post infection.

### Western blot analysis

Cells were washed twice in ice-cold PBS, suspended in the lysis buffer containing 50 mM Tris-HCl pH 7.4, 300 mM NaCl, 1% NP-40, 50 mM NaF, 1 mM NaVO4, 1 mM PMSF and 1x protease inhibitor cocktail (Calbiochem, La Jolla, CA), and incubated on ice for 20 min. After centrifugation at 20,000*g* at 4°C for 20 min, the supernatants were collected and saved as aliquots. The protein concentration was determined with a Bio-Rad protein assay kit (Bio-Rad, Hercules, CA). The lysates were subjected to 12% SDS-polyacrylamide gel electrophoresis and the proteins transferred to polyvinylidene fluoride membranes (Amersham Biosciences). ORP4, HCV core, and GFP proteins in the cell lysates were detected by immunoblotting the membrane with anti-mouse anti-Flag M2 (Sigma), anti-mouse anti-core (Thermo Scientific, Rockford, IL), and anti-rabbit anti-GFP (Clontech, Mountain View, CA) antibodies, respectively. The secondary antibody was conjugated to horseradish peroxidase and the proteins were visualized using the ECL system (Amersham Biosciences, Pittsburgh, PA, USA).

### Immunofluorescence staining

Cells were grown on polylysine-coated cover slips and transfected with the indicated plasmids or treated with the lipid synthesis inhibitors as described above. For endogenous ORP4 expression, cells were washed twice with PBS, fixed in 4% paraformaldehyde, permeabilized with 0.3% Triton X-100 for 5 min, and blocked in 2.5% BSA for 30 min. Cells were incubated with FITC-conjugated donkey anti-Flag antibody (Santa Cruz Biotechnologies, Santa Cruz, CA) at room temperature for 1 hr and washed three times with PBS to visualize ORP4 expression. For ORP4 and endoplasmic reticulum (ER) co-localization staining, Huh 7.5 cells were grown on cover slips and transfected with pCMV-flag-ORP4 and pDsRed2-ER, cultured for 48 hr, and then washed twice with PBS, fixed in 4% paraformaldehyde, permeabilized with 0.2% Triton X-100 for 30 min, and blocked in 3% BSA for 1 hr. The cells were then incubated with anti-mouse flag monoclonal antibody (Sigma) for 1 hr followed by FITC-conjugated donkey anti-goat secondary antibody (Santa Cruz Biotechnologies) at room temperature for 1 hr. For Oil Red O staining, cells were stained with freshly prepared and filtered 0.5% Oil Red O (Sigma) for 15 min, rinsed with 60% isopropanol, and washed once with PBS. Nuclei of these cells were stained with DAPI (10 ng/ml), rinsed with PBS, and mounted with 50% glycerol on the slide glass. Cells were visualized with an immunofluorescence microscope, as described previously [28].

### Cell-based HCV NS5B activity assay

A RIG-I-dependent cell-based HCV NS5B activity assay was performed as described previously [29]. The assay uses HCV NS5B to synthesize RNA that is recognized by the RIG-I protein to activate expression of firefly luciferase driven by either an IFNβ promoter or an NFκB promoter. pTK-Luc expressing *Renilla* luciferase under the control of the RIG-I insensitive thymidine kinase promoter was used as a transfection and toxicity control. Briefly, 293T cells were plated in a 96-well plate and transfected with 30 ng each of pCMV-myc-NS5B, pUNO-hRIG, pNFκB-Luc, and pTK-Luc and an indicated amount of pCMV-flag-ORP4, or pCMV-flag-ORP4S using Lipofectamine 2000 (Invitrogen). The pCMV-myc cloning vector was used to equalize the total amount of the DNA transfected among experiments. The cells were harvested 24 hr post transfection for quantification of both firefly luciferase (FF) and *Renilla* luciferase (Ren) activity using the Dual-Glo Luciferase Assay system (Promega). The FF/Ren ratio was calculated and used to express the relative HCV NS5B activity.

### Cholesterol assay

Cholesterol levels in cell culture supernatants and cells were measured using an Amplex Red Cholesterol Assay Kit (Invitrogen). Briefly, cells were cultured and transfected in a 24-well plate. Forty-eight hours post transfection, the cell culture supernatants were collected and cell debris was removed by a brief centrifugation. Clarified supernatants were saved and used for determination of secreted cholesterol. Cells were then washed with ice-cold PBS and lysed in 100 µl lysis buffer (1% Triton X-100 and 1X Roche protease inhibitor cocktail in PBS). The cell lysates were centrifuged at 12,000*g* for 20 min and the concentration of lysates was determined using a BioRad DC kit. Lysates were then diluted in the 1X reaction buffer from an Amplex Red Cholesterol Assay Kit. Ten microliters of the clarified cell culture supernatants or diluted cell lysates were used to determine the cholesterol level using the Amplex Red cholesterol assay kit. Absolute cholesterol levels were calculated based on standards analyzed in parallel in the assays.

### Data analysis

All values are expressed as means ± SD of triplicate experiments. All comparisons were made based on the control using a two-tailed Student’s *t*-test. A *p* value of < 0.05 was considered statistically significant (*), and *p* < 0.01 highly significant (**).

## Results

### Identification of cellular proteins in the HCV replication complex

**Figure 1:**
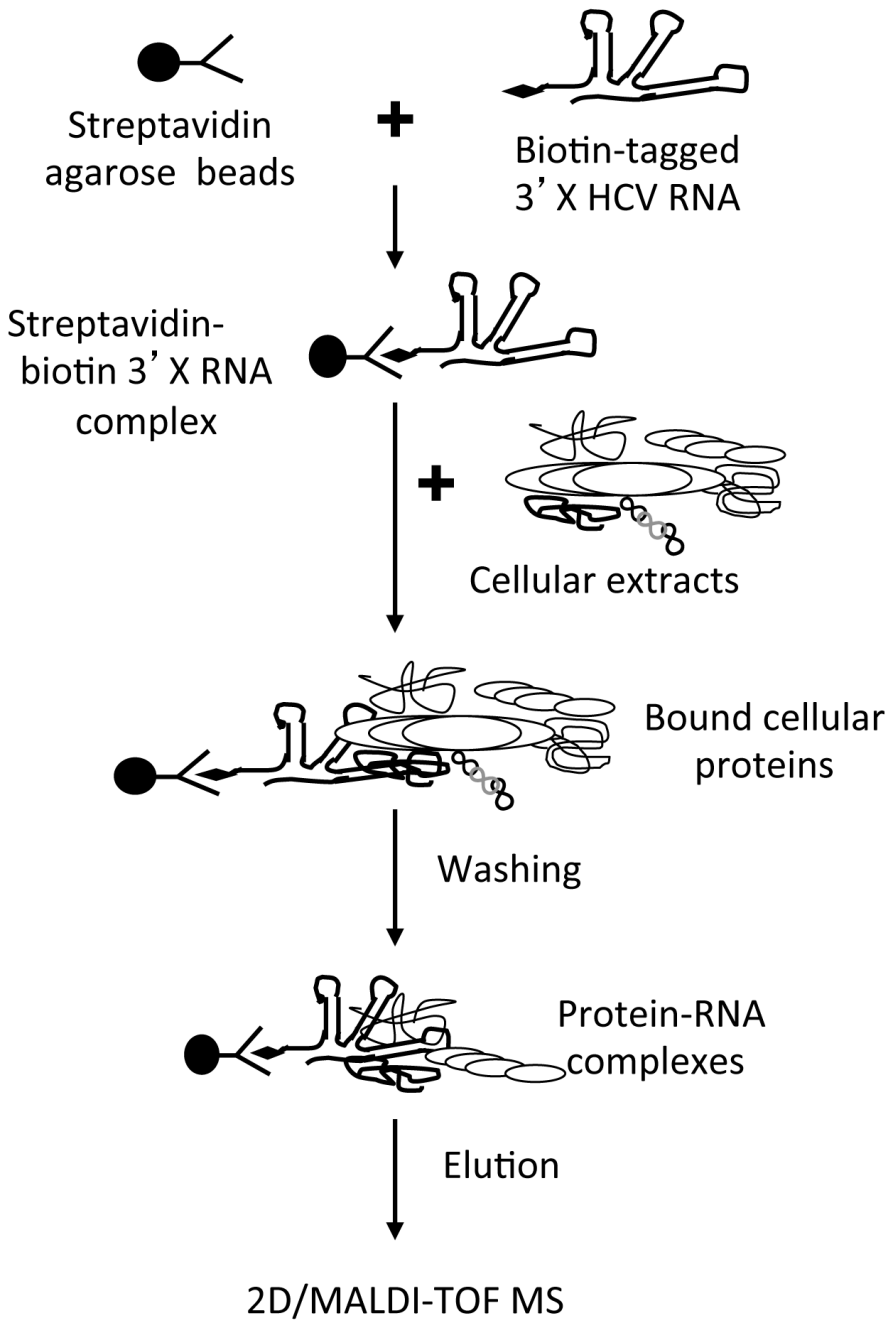
Scheme of the RNA affinity chromatography-2D/MASS strategy. Cell extracts were prepared from 293T, Huh7.5, and Clone B cells and were added to streptavidin agarose beads that were pre-conjugated with biotinylated HCV 3’X RNA. The beads were extensively washed to remove unbound proteins, and the proteins that either directly or indirectly bound to RNA were then eluted and analyzed by 2D/MASS.

To isolate cellular proteins present in the HCV replication complex, a biotin-3’X RNA was generated by *in vitro* transcription, and RNA affinity chromatography was performed using cell extracts from hepatocyte Huh7.5 cells (Figure **1**). Proteins from hepatocytic Clone B cells that express HCV subgenomic RNA and nonstructural proteins and the human embryonic kidney cells that do not express HCV proteins were processed in parallel [30]. The conjugating efficiency of the biotin-3’X RNA to streptavidin coated-agarose beads was verified by comparing the bound RNA with input RNA on a 5% polyacrylamide gel (data not shown). The biotin-3’X RNA-streptavidin agarose beads were incubated with cell extracts, and excess yeast tRNA was included in the mixture to increase the binding stringency and specificity. The agarose beads were then thoroughly washed, and the associated proteins were eluted and analyzed by 2D gel electrophoresis. Proteins that showed differences in staining intensity among Huh7.5, hepatocytic clone B and 293T cells were quantified, excised, digested with trypsin, and then analyzed by MALDI-TOF.

**Figure 2:**
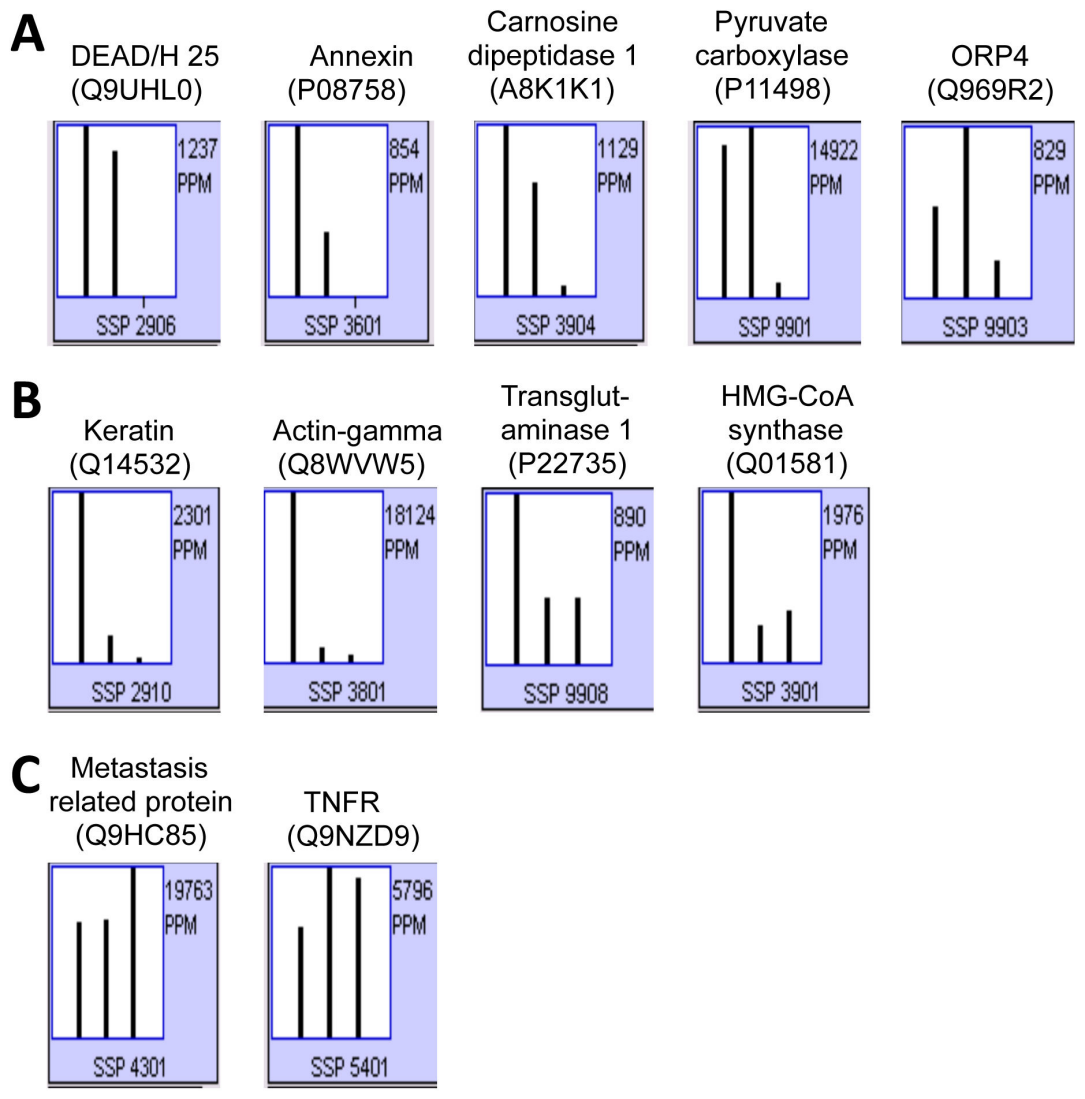
Relative levels of the HCV 3’ X-associated proteins in Huh7.5, Clone B, and 293T cells. HCV 3’ X-associated proteins were fractionated on the 2D gel. The level of each protein (spot) was quantified using the PDQuest software, expressed in parts per million (ppm) and compared among Huh7.5, Clone B, and 293T cells (from left to right in each histogram). The identity of each protein revealed by MASS was given on the top of the histogram along with the SwissProt accession number in parenthesis and the standard spot (SSP) number at the bottom of the histogram.

Eleven cellular proteins were identified to bind 3’ X (Table **1**). Based on the relative levels among three cell lines, these proteins were divided into three groups: (1) five proteins that were detected in Huh7.5 cells and Clone B cells: DEAD/H25, annexin, carnosine dipeptidase 1, pyruvate carboxylase, and oxysterol binding protein-related protein 4 (ORP4) (Figure **2A**); (2) four proteins that were detected at a much higher level in Huh7.5 cells: keratin, actin-γ, transglutaminase 1, 3-hydroxy-3-methylglutaryl-Coenzyme A (HMG-CoA) synthase (Figure **2B**); and (3) two proteins that were detected in all three cell lines: metastasis-related protein and tumor necrosis factor receptor (TNFR) (Figure **2C**). We note that HMG-CoA synthase is known to control HCV replication through regulation of cholesterol and fatty acid biosynthesis [31]. Furthermore, small molecule inhibitors of HMG-CoA reductase such as lovastatin inhibit HCV replication in HCV full-length and subgenomic replicon systems [31-33] have provided strong evidence to support the involvement of the cholesterol biosynthetic pathway in HCV replication. Identification of HMG-CoA synthase provided important validation for the present strategy for host proteins involved in HCV replication. The known functions and possible roles of all other proteins in HCV replication were briefly summarized in Table **1**. Most relevant to this study, ORP4 is a cytoplasmic receptor for oxysterols, the oxygenated derivatives of cholesterol known to down-regulate HMG-CoA reductase synthesis; it belongs to the protein family called OSBP-related protein (ORP) and is known to be significant for cholesterol and lipid metabolism [34-37].

**Table 1:** Host proteins that were detected in the HCV 3’ X complex identified by 2D/MASS.

***SwissProtaccession#***	***ProteinName***	***Mass(kDa***)	***pI*^*a*^**	***Percentpeptidecoverage***	***Z*^*b*^*score***	***Knownfunctionand/orpossibleroleinHCVreplication*^*c*^**
P08758	Annexin 5	35.96	4.9	27	1.33	Anti-apoptosis, signal transduction (?)
A8K1K1	Carnosine dipeptidase 1	56.79	5.1	15	2.43	Proteolysis (?)
P11498	Pyruvate carboxylase	130.37	6.4	51	2.4	Gluconeogenesis, lipid biosynthesis (?)
Q969R2	KIAA1664 (ORP4)	100.75	6.1	9	2.43	Lipid metabolism, vesicle transport, signaling (?)
Q9UHL0	DEAD/H box polypeptide 25	55.06	5.9	7	1.82	RNA helicase (?)
Q8WVW5	Actin gamma	40.83	5.8	40	2.43	Cell motility (?)
Q01581	HMG-CoA synthase	57.85	5.2	21	2.43	Cholesterol and isoprenoid syntheses, lipid metabolism (25)
P22735	Transglutaminase 1	90.12	5.7	10	2.43	Protein modification, cell envelope biogenesis (?)
Q14532	Keratin	51.78	4.8	17	2.43	Epidermis development (?)
Q9NZD9	Tumor necrosis factor receptor	14.72	5.7	45	0.75	Apoptosis, signal transduction (?)
Q9HC85	Metastasis-related protein	10.40	5.4	59	1.27	Unknown (?)

a) pI is the isoelectric point of each protein.

b) The Z score calculated by ProFound® search engine and is an indicator of the search result quality. It is estimated when the search result is compared against an estimated random match population and is the distance to the population mean in units of standard deviation.

c) Known functions of each protein are derived from the literature and used to indicate their possible roles in HIV replication, which are marked as in the table.

### ORP4 expression and subcellular localization in hepatocytes

**Figure 3:**
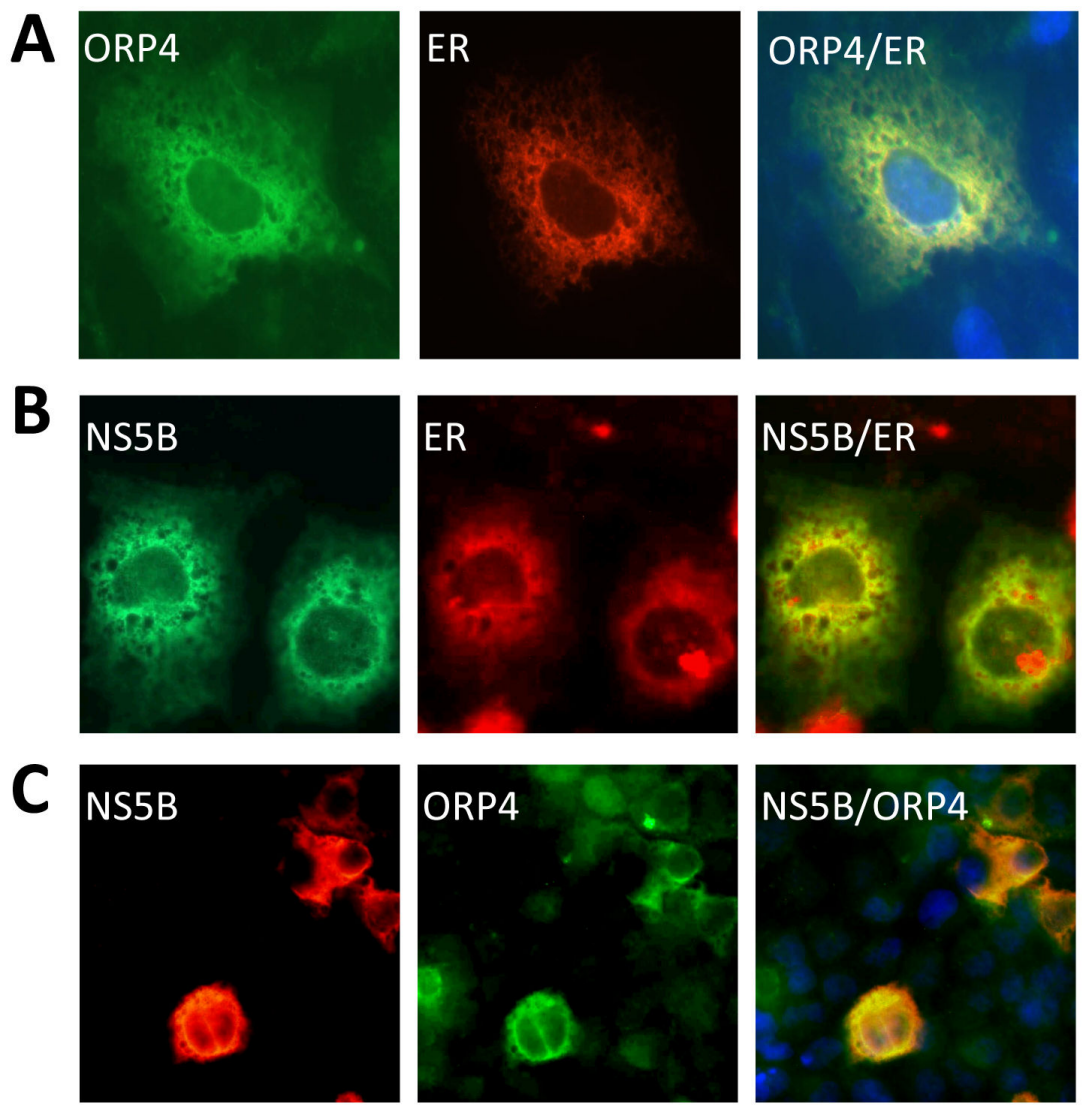
Expression and subcellular localization of ORP4. Huh7.5 cells were transfected with pCMV-flag-ORP4 and pDsRed2-ER (**A**), pCMV-myc-NS5B and pDsRed2-ER (**B**), or pCMV-flag-ORP4 and pCMV-myc-NS5B (**C**) expressing an ER-targeted red fluorescence protein (RFP). Forty-eight hours post-transfection, cells were washed, fixed with 4% PFA, permeabilized and stained with anti-flag antibody for ORP4 or anti-myc for NS5B, followed by FITC-conjugated IgG for ORP4 (**A** & **C**), Alexa-fluor 488 IgG for NS5B (**B**) or PE-conjugated IgG for NS5B (**C**). Appropriate isotype-matched IgG antibodies were included to ensure the antibody specificity. Nuclei of the cells were stained with DAPI. Representative micrographs of stained cells were taken using a Zeiss M200 fluorescence microscope. The data were representative of three independent experiments.

Oxysterol-binding protein-related protein 4 (ORP4) is a cytoplasmic receptor for oxysterols (oxygenated derivatives of cholesterol known to down-regulate HMG-CoA reductase expression) that belongs to a large family of OSBP-related proteins (ORPs) known to be involved in cholesterol trafficking and/or signaling [34-37]. To investigate the role of ORP4 in HCV replication, we first determined ORP4 expression and localization in hepatocytes by immunofluorescence staining. Little endogenous ORP4 protein expression was detected in Huh7.5 cells, as well as in human primary hepatocytes (data not shown). The negative results may be due to the inability of the only available antibody to recognize the ORP4 protein in the context of immunofluorescence staining or low ORP4 expression in these cells that the antibody failed to detect. RT-PCR with ORP4-specific primers was able to detect ORP4 mRNA in these cells but the message was in low abundance compared to the housekeeping gene β-actin control (data not shown). Next, we investigated subcellular localization of ORP4 in Huh7.5 cells through ectopic expression using pCMV-flag-ORP4. As HCV replication is associated with membranes derived from the endoplasmic reticulum (ER), we determined whether ORP4 protein localized to the ER. To visualize the ER, an ER-targeted red fluorescence protein expression plasmid pDsRed2-ER was also included in the transfection. ORP4 and RFP localized extensively, suggesting that ORP4 likely resides in ER-associated membranes (Figure **3A**).

Several RNA-protein binding assays were attempted to determine whether ORP4 directly bound to the HCV 3’ X region. To our surprise, none of the assays indicated a direct interaction between ORP4 and the 3’X RNA (data not shown). This unexpected finding led us to examine the other possibility, that is that the detection of ORP4 in the HCV 3’ X-containing replication complex is due to association of ORP4 with other proteins in the HCV replication complex. To address this, we expressed HCV NS5B, an indispensable component in the HCV 3'X-containing replication complex, and compared its localization to that of ORP4 in Huh7.5 cells transfected with pCMV-myc-NS5B and pDsRed2-ER, or pCMV-flag-ORP4 and pCMV-myc-NS5B. As expected, NS5B was detected in and co-localized with ER-associated membranes (Figure **3B**). Importantly, Co-expressed NS5B and ORP4 also showed an overlapping staining pattern (Figure **3C**). Taken together, these results support the notion that ORP4 is involved in HCV replication, likely through its association with other proteins in the HCV 3X-containing replication complex (Table **1**).

### Inhibition of HCV replication by ORP4 expression

**Figure 4:**
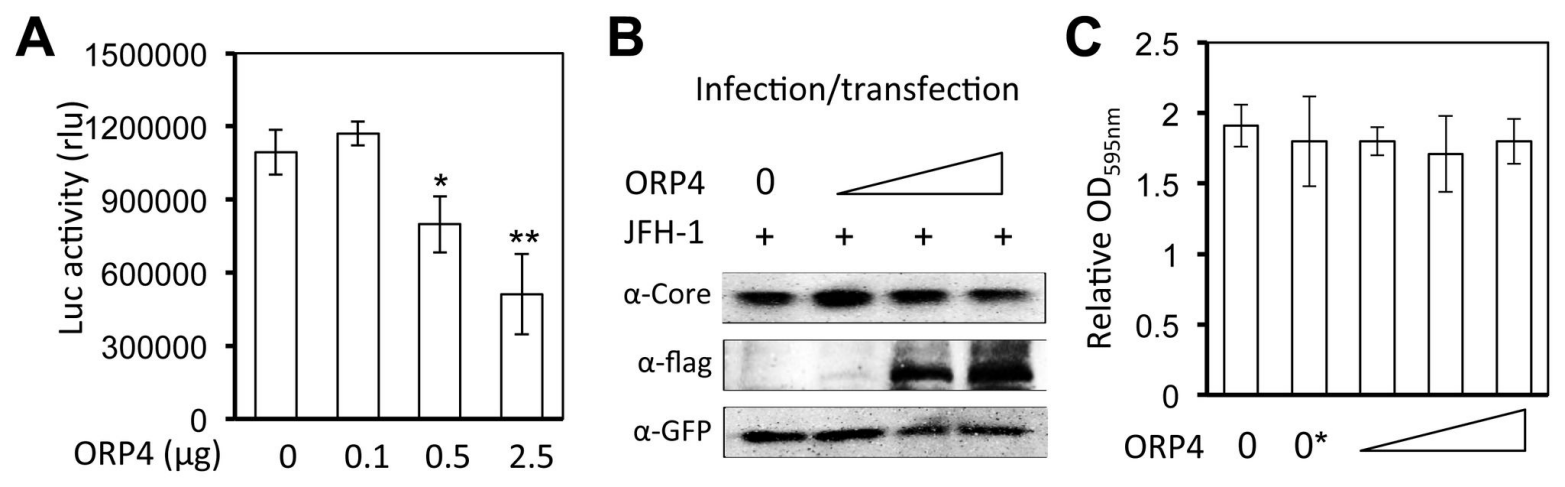
Inhibition of HCV replication by ORP4 expression. **A**. HCV subgenomic replicon RLuc cells were cultured in a 6-well plate and transfected with an indicated amount of pCMV-flag-ORP4 expression plasmid. pcDNA3, the cloning backbone vector, was used to equalize the total amount of plasmid DNA among the transfections; pCMV-βGal was included in each transfection to normalize the transfection variations. Cell lysates were prepared 48 hr post-transfection for the β-Gal activity and luciferase activity assay. The data are Means ± SD of triplicates and are representative of at least three independent experiments. **B** & **C**. Huh7.5 cells were cultured in a 24-well plate, first infected with HCV JFH-1 (0.5 MOI), then transfected with an increasing amount of pCMV-flag-ORP4 expression plasmid (62.5 ng, 250 ng, and 1 µg). An equal amount of pc3.GFP expression plasmid was included in all transfection to ensure a comparable transfection efficiency. Cells were harvested 3 days post infection for cell lysates and Western blotting using an anti-HCV core, Flag, or GFP antibody (**B**), or for MTT assay (**C**). 0* in panel C indicated that these cells were not transfected the ORP4 expression plasmid but infected with JFH-1. The data are Means ± SD of triplicates and/or are representative of at least three independent experiments.

Next, we determined the relationship between ORP4 expression and HCV replication by ectopic expression of ORP4. Considering that hepatocytes have low levels of ORP4 protein and mRNA, we decided to determine the effect of ectopic ORP4 expression on HCV replication. HCV replication was assessed using a stable subgenomic replicon that expresses *Renilla* luciferase, RLuc [26]. Compared with the control, ORP4 expression in the HCV replicon cells led to a significant and dose-dependent decrease in the luciferase reporter gene activity (Figure **4A**). Next, we infected Huh7.5 cells with JFH-1 and then transfected the cells with the ORP4 expression plasmid. pc3.GFP was included to ensure a comparable transfection efficiency. ORP4 expression resulted in a dose-dependent inhibition of HCV core expression (Figure **4B**). ORP4 expression exhibited little effect on cell proliferation and survival (Figure **4C**), suggesting that the inhibitory effect is not due to any adverse effect of ORP4 on these cells. Similar results were obtained in cells that were first transfected with ORP4, followed by infection with JFH-1 (data now shown).

### Involvement of the oxysterol-binding domain in ORP4-mediated HCV inhibition

**Figure 5:**
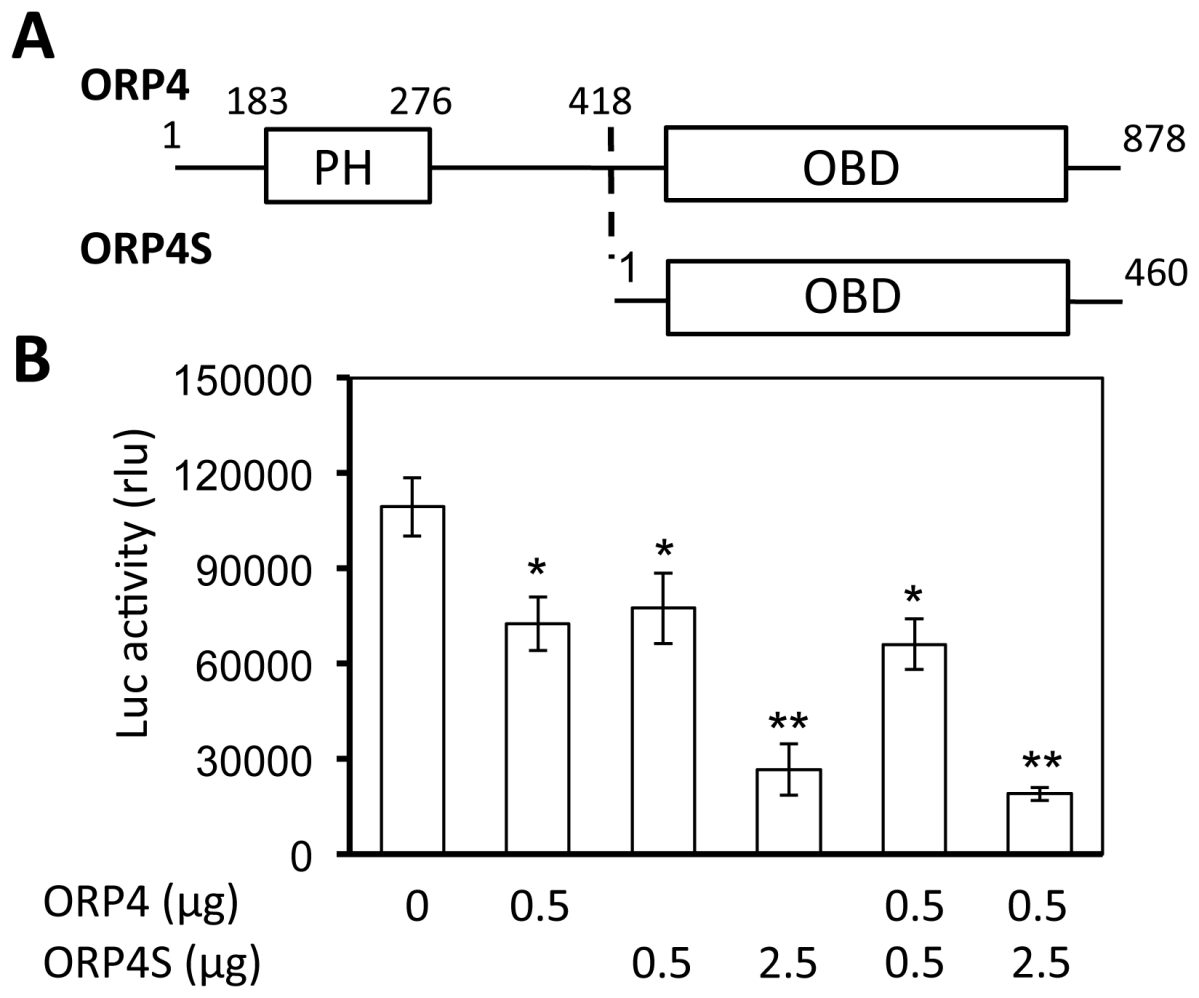
Inhibition of HCV replication by an ORP4 variant expression. **A**. ORP4 has a pleckstrin-homolog domain (PH) for protein-protein interaction and an oxysterol-binding domain (OBD) for oxysterol binding and transportation. ORP4S is a naturally occurring ORP4 splice variant and only contains the OBD domain-containing C terminus of ORP4. **B**. HCV RLuc cells were transfected with ORP4, ORP4S, or both. pcDNA3 was used to equalize the total amount of plasmid DNA among the transfections; pCMV-βGal was included in all transfection to normalize the transfection variations. Cell lysates were prepared 48 hr post transfection for the β-Gal activity and luciferase activity assay. The data are Means ± SD of triplicates and are representative of at least three independent experiments.

ORP4 contains a N-terminal pleckstrin-homology domain (PH) and a C-terminal oxysterol-binding (OSBD) domain which are involved in protein-protein interaction and sterol-binding and transportation, respectively (Figure **5A**). We took advantage of the naturally occurring ORP4 variant ORP4S, which contains the OSBD but not the PH domain [38,39], and evaluated its effect on HCV replication in cells expressing RLuc. Similarly to ORP4, ORP4S expression inhibited HCV replication and appeared to be more potent than ORP4 (Figure **5B**). However, co-expression of ORP4 and ORP4S did not lead to greater inhibition compared to ORP4S expression alone. These results show that the OSBD domain is directly involved in ORP4-mediated HCV inhibition, and suggest that a proper level of sterol binding and transportation is important for HCV replication.

### ORP4 interaction with HCV NS5B

**Figure 6:**
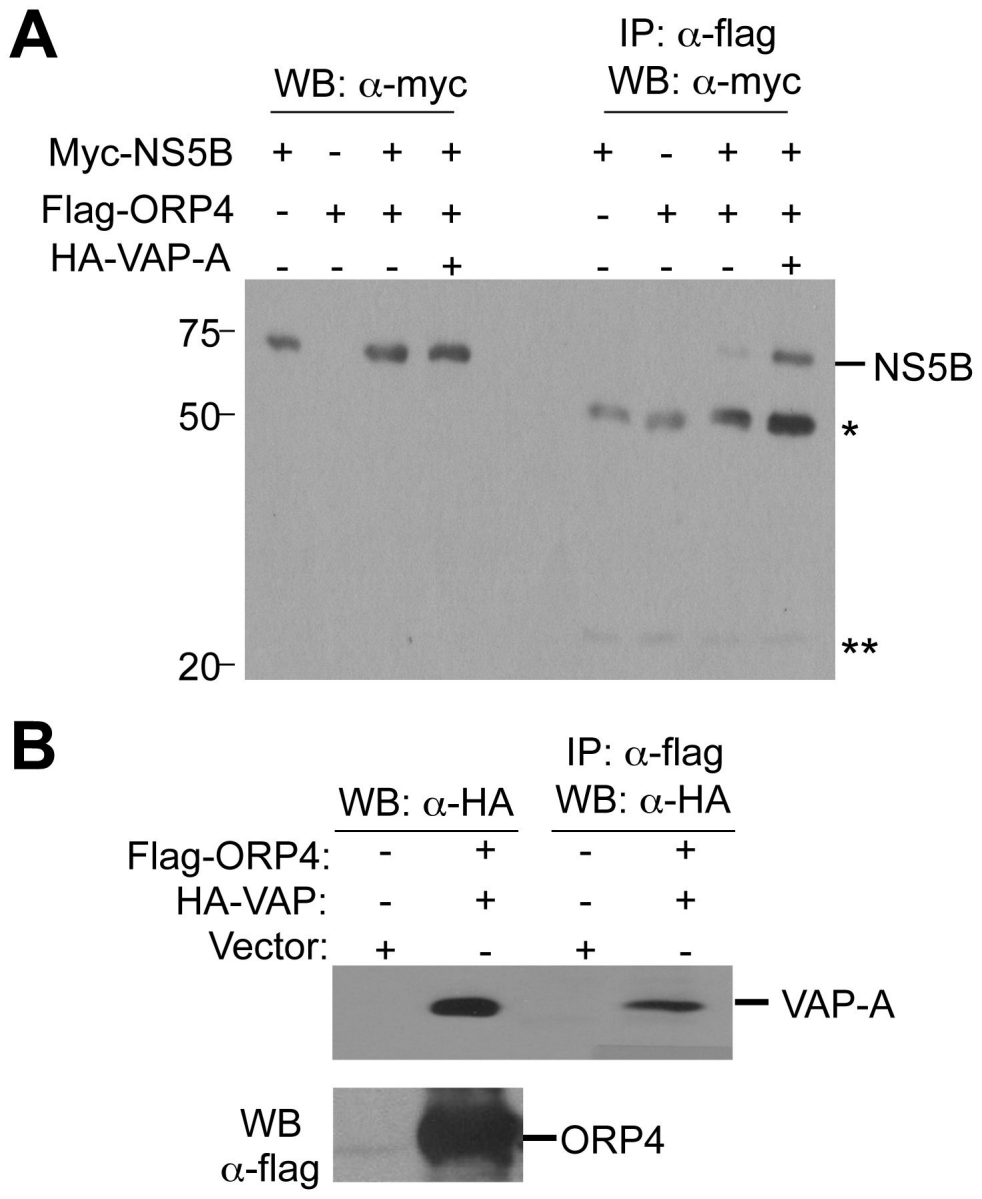
ORP4 interaction with NS5B. **A**. 293T cells were transfected with pCMV-myc-NS5B alone, pCMV-flag-ORP4 alone, pCMV-myc-NS5B and pCVM-flag-ORP4, or pCMV-myc-NS5B and pCMV-flag-ORP4 and pCMV-HA-hVAP. Cell lysates were prepared 48 hr post transfection for Western blotting with α-myc antibody, or immunoprecipitated with anti-flag antibody followed by Western blotting with α-myc antibody. *: reactive IgG heavy chain; **: reactive IgG light chain. The data were representative of three independent experiments. **B**. 293T cells were transfected with pCMV-flag or pCMV-flag-ORP4 and pCMV-HA-hVAP. Cell lysates were prepared as above, for Western blotting with anti-HA antibody, or immunoprecipitated with α-flag antibody followed by Western blotting with anti-HA antibody.

We sought to identify a possible mechanism for the effects of ORP4. ORP4 interacts with hVAP-A [40], which is known to interact with viral proteins NS5A and NS5B and promote the formation of HCV replication complex on lipid rafts [41-43]. Thus, we next determined whether ORP4 formed a complex with HCV NS5B by transfecting 293T cells with plasmids encoding flag-ORP4, myc-NS5B, flag-ORP4 and myc-NS5B, or Flag-ORP4 and myc-NS5B and HA-hVAP-A. Cell extracts were immunoprecipitated with an anti-flag antibody for ORP4 and Western blotted with an anti-myc antibody for NS5B. NS5B was detected in the ORP4 immunoprecipitates (Figure **6A**). Consistent with a role for hVAP-A [40], hVAP-A co-expression enhanced NS5B-ORP4 co-immunoprecipitation (Figure **6B**). These results support a model where ORP4 is associated with the HCV replication complex through interaction with NS5B and/or hVAP-A.

### Inhibition of HCV NS5B activity by ORP4 expression

**Figure 7:**
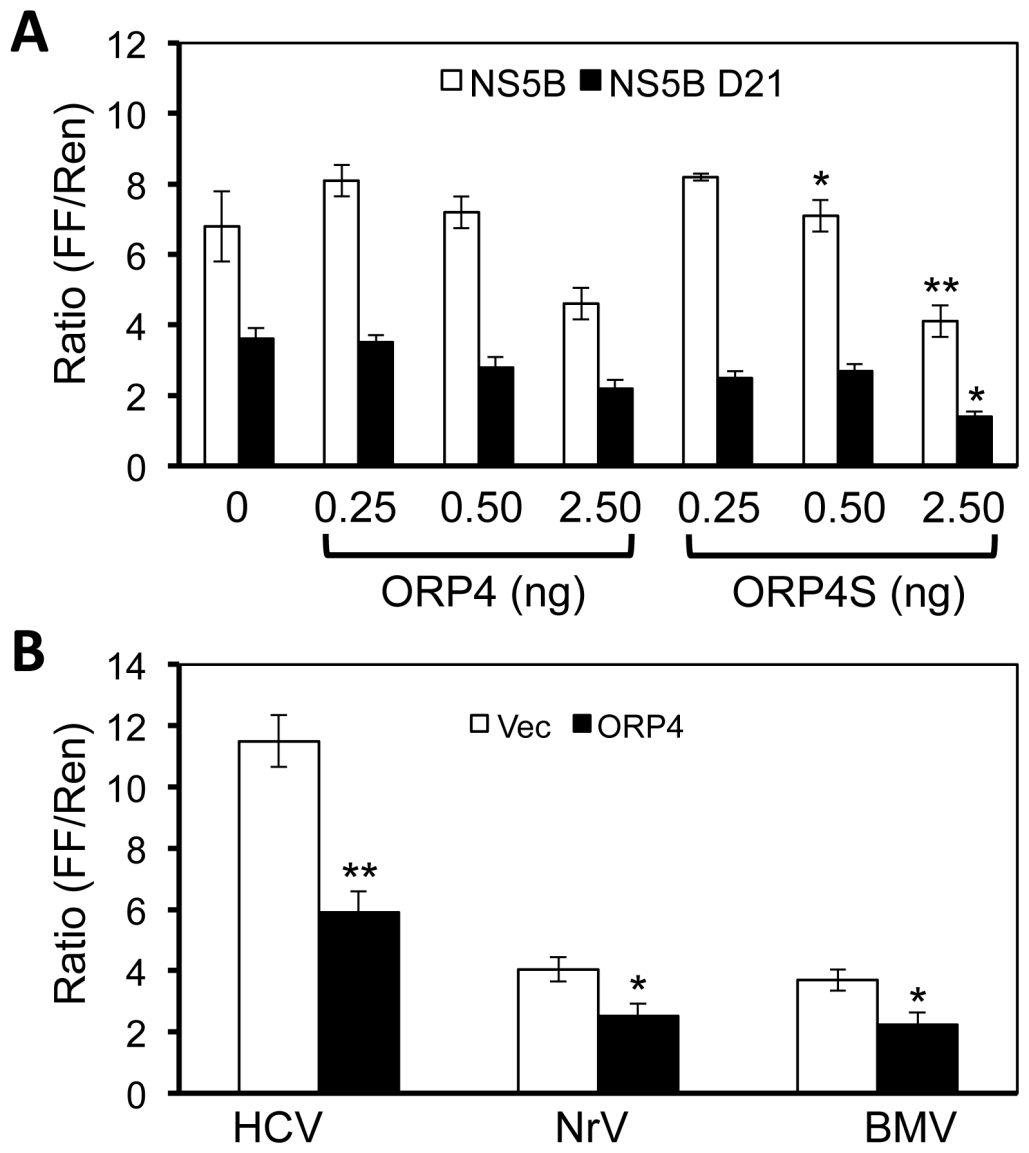
Inhibition of HCV NS5B polymerase activity by ORP4 expression. **A**. 293T cell were plated and cultured in a 96-well plate and transfected with 30 ng each of pUNO-hRIG, pNFκB-Luc, and pCMV-myc-NS5B (open bar), or pCMV-myc-NS5B Δ21 (closed bar) containing the deletion of the 21 amino acid catalytic domain of NS5B, and increasing amounts of pCMV-flag-ORP4 or pCMV-flag-ORP4S as indicated. *Renilla* luciferase driven by the RIG-I-insensitive thymidine kinase promoter (pTK-Luc) was included in all transfections as a transfection and toxicity control. pCMV-myc cloning vector was used to equalize the total amount of the DNA transfected among all transfections. The cells were harvested 24 hr post transfection for both firefly luciferase (FF) and *Renilla* luciferase (Ren) activity assays. The FF/Ren ratio was calculated and used to express the relative HCV NS5B activity. **B**. 293T cells were plated and transfected as described above except for that the same amount of pCMV-myc-ORP4 (2.5 ng, closed bar) was transfected with 30 ng of the plasmid expressing HCV NS5B polymerase (HCV), Norovirus polymerase (NrV), or Brome mosaic virus polymerase (BMV). pCMV-myc cloning vector (Vec, open bar) was used as the transfection control.

The co-localization and interaction between ORP4 and NS5B could lead to inhibition of NS5B enzymatic activity and as a result, lead to inhibition of HCV gene expression and replication. To address this possibility, we performed a cell-based NS5B enzymatic activity assay [29]. In this assay, any RNA that is synthesized by NS5B will be recognized by RIG-I, which in turn will activate downstream signaling and result in the expression of the RIG-I-dependent reporter genes. Activity of the reporter genes have been validated for measurement of the NS5B activity and inhibitors specific to NS5B [27]. We transfected 293T cells with plasmids expressing HCV NS5B, RIG-I, a RIG-I-dependent firefly luciferase reporter, and increasing amounts of plasmid to express ORP4. A RIG-I-independent *Renilla* luciferase reporter plasmid was included to control for the transfection and cytotoxicity. The results showed that the relative firefly luciferase activity gradually decreased with ORP4 expression (Figure **7A**). Activation of RIG-I by exogenously transfected agonists did not exhibit an inhibitory effect on reporter gene expression in the presence of ORP4, suggesting that ORP4 did not act through RIG-I (data not shown). Furthermore, HCV NS5B Δ21 containing the deletion of the 21 amino acid transmembrane helix exhibited reduced inhibition by ORP4, suggesting that proper membrane association by NS5B facilitates interaction with ORP4. Furthermore, ORP4S also showed similar inhibitory effects on NS5B activity in the same assay.

To determine whether the inhibitory effects of ORP4 were specific to HCV NS5B, we compared HCV NS5B with polymerases of two other RNA viruses Norovirus and Brome mosaic virus. Compared to the vector control, ORP4 showed minor inhibitory effects on those two RNA polymerases compared to HCV (Figure **7B**). Taken together, these results showed that ORP4 preferentially inhibits HCV NS5B activity.

### The relationship among ORP4 expression, lipid metabolism and HCV replication

**Figure 8:**
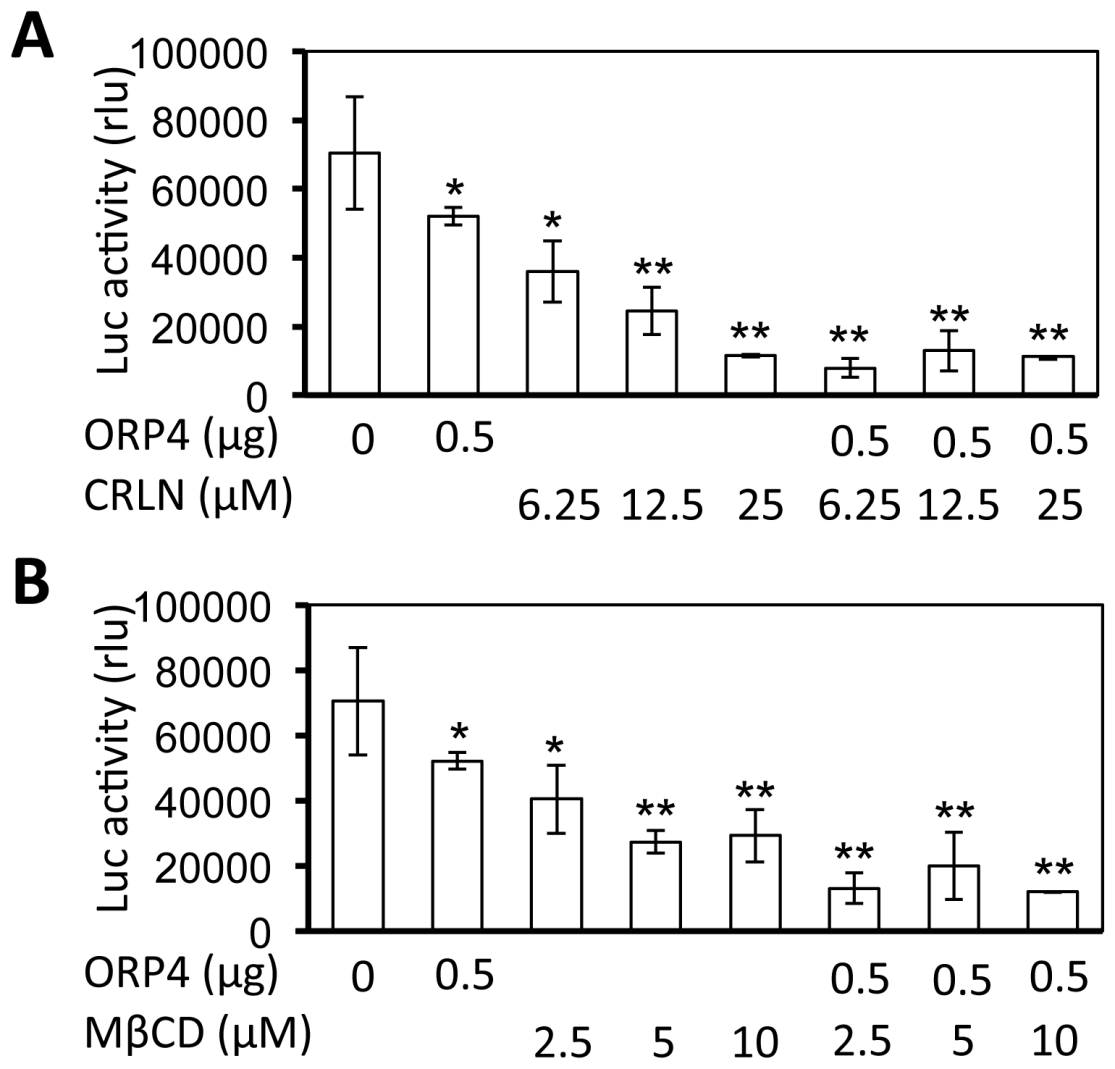
Inhibition of HCV replication by ORP4, lipid synthesis inhibitor, and/or lipid sequestering agent. HCV RLuc cells were transfected with the ORP4 expression plasmid. pcDNA3 was used to equalize the total amount of plasmid DNA among the transfections, and pCMV-βGal was included in each transfection to normalize the transfection variations. The transfected cells were cultured for 48 hr and then treated with lipid synthesis inhibitors cerulenin (CRLN) (**A**), or lipid sequestering agent methyl-β-cyclodextrin (MβCD) (**B**) for 24 hr. Cell lysates were prepared for the β-Gal activity and luciferase activity assay. The data are Means ± SD of triplicates and are representative of at least three independent experiments.

HCV replication is tightly regulated by lipid metabolism [44], and the OSBD-containing proteins are involved in the transport and metabolism of cholesterol, oxysterols and other lipids at a variety of intracellular membranes [45]. Thus, we next determined the relationship between ORP4 expression, lipid metabolism and HCV replication. We took advantage of the lipid synthesis inhibitor cerulenin (CRLN), a potent inhibitor of fatty acid synthase [46]. We treated the HCV replicon cells HCV RLuc with CRLN, alone or in combination with ORP4 expression and determined their effects on HCV replication. As expected, treatment of the replicon cells with CRLN inhibited HCV replication in a dose-dependent manner (Figure **8A**). CRLN treatment of the cells that were transfected with ORP4 appeared to have greater inhibition of HCV replication than ORP4 expression alone, or CRLN treatment alone. Similar results were obtained with another lipid synthesis inhibitor 25-hydroxycholesterol (25-HC) [47] (data not shown). We also evaluated the effect of methyl-β-cyclodextrin (MβCD) on HCV replication. MβCD extracts cholesterol from the plasma membrane and eventually intracellular membranes, and disrupts lipid rafts [48]. Similarly to CRLN, MβCD treatment alone inhibited HCV replication and appeared to achieve a higher level of HCV inhibition in cells expressing ORP4 expression (Figure **8B**). Taken together, these results demonstrate that ORP4 expression in combination with a lipid synthesis inhibitor or cholesterol sequestering agent synergize to inhibit HCV replication, suggesting that alterations of lipid synthesis or intracellular lipid distribution might be involved.

### Alteration of lipid droplet formation by ORP4 expression

**Figure 9:**
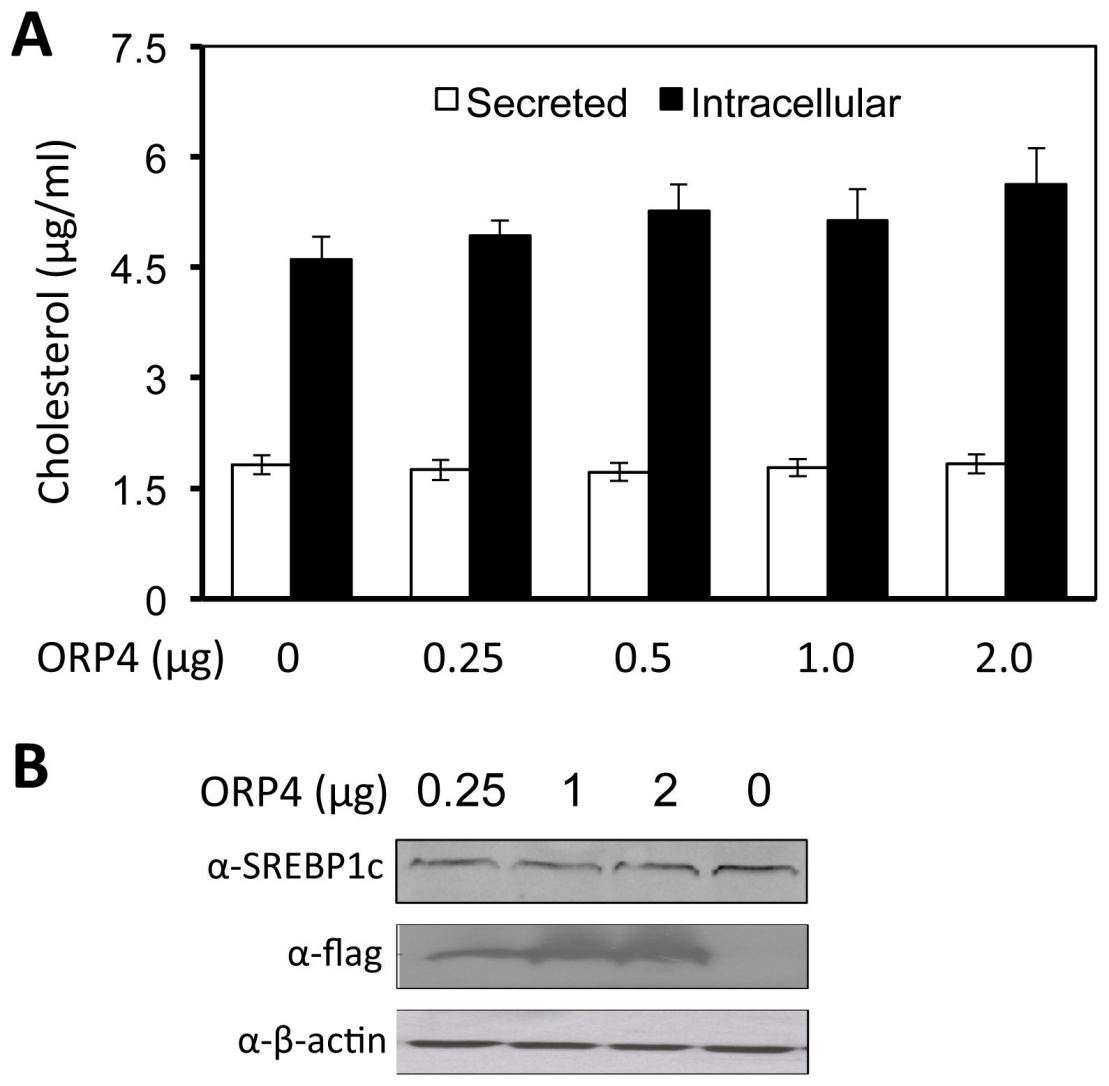
ORP4 expression had no effects on cholesterol production. **A**. Huh7.5 cells were transfected with the indicated amount of ORP4 expression plasmid. Forty-eight hours post transfection, cell culture supernatants were collected, while the cells were harvested for cell lysates. Cell culture supernatants and cell lysates were used to determine secreted cholesterol (open bar), the total intracellular cholesterol (closed bar), respectively. Standards were used to calculate the absolute concentration of cholesterol in both samples. The data are Means ± SD of triplicates and are representative of at least three independent experiments. **B**. Similar transfections were performed, the cell lysates were analyzed for SREBP1c and ORP4 expression by Western blotting. β-actin was included as an equal loading control.

We first determined the effect of ORP4 on lipid synthesis. We transfected Huh7.5 cells with the ORP4 expression plasmid, harvested the cells, prepared cell lysates and measured the total cholesterol (intracellular or total cholesterol) using a cholesterol oxidase-based kit. We also collected cell culture supernatants and measured cholesterol levels in cell culture media (secreted total cholesterol). The transfection efficiency of ORP4 was estimated to be more than 90%, as determined by immunofluorescence staining (data not shown). ORP4 expression had no significant effect on either total or secreted cholesterol levels (Figure **9A**). In addition, unlike oxysterol-binding protein (OSBP) [49], ORP4 expression did not significantly alter expression of sterol regulatory element-binding protein 1c (Figure **9B**), an important regulator of fatty acid metabolism [50]. Furthermore, experiments in HCV-infected Huh7.5 cells provided similar results (data not shown).

**Figure 10:**
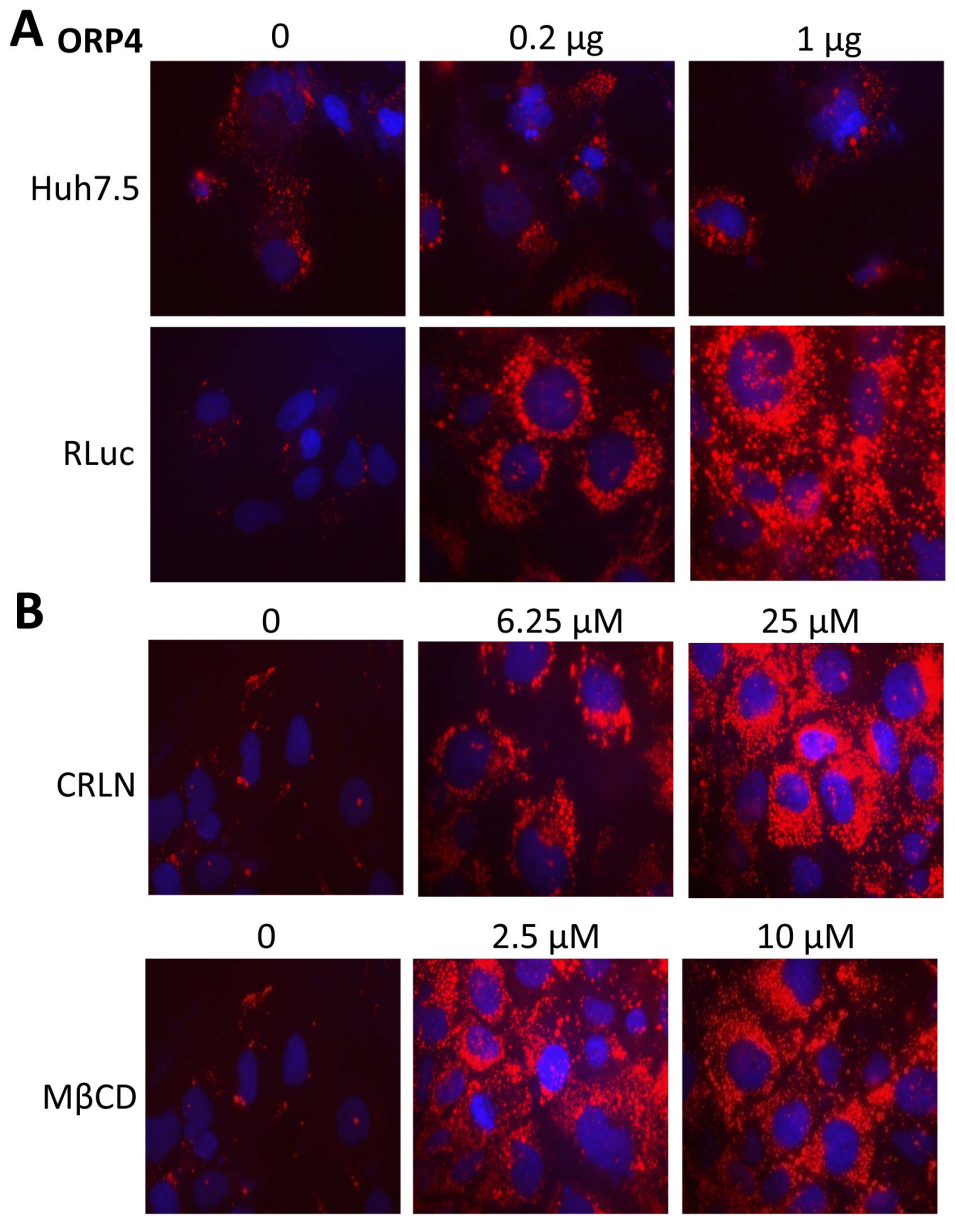
Formation of lipid droplets induced by ORP4 and lipid synthesis inhibitors or lipid sequestering agents. **A**. Huh7.5 or HCV RLuc cells were cultured on coverslips and transfected with 0, 0.2, or 1 µg ORP4 expression plasmid. pcDNA3 was used to equalize the total amount of plasmid DNA among the transfections. The transfected cells were processed to determine lipid droplet formation using Oil Red O staining 48 hr post transfection and microscopic imaging. **B**. HCV RLuc cells were treated with CRLN, or MβCD at indicated concentrations and processed for Oil Red O staining and lipid droplet formation.

We next examined the effect of ORP4 on lipid distribution. We transfected ORP4 expression plasmid into both naïve Huh7.5 and Huh7.5-derived HCV replicon cells RLuc and performed the Oil Red O staining of these cells for lipid droplets. There were only a few lipid droplets in both Huh7.5 and HCV RLuc cells in the absence of ORP4 over-expression (**top panel,** Figure **10A**); ORP4 expression did not appear to change lipid droplet formation in Huh7.5 cells. In contrast, ORP4 expression led to a dramatic increase in lipid droplets in HCV RLuc cells that was dose-dependent (**low panel,** Figure **10A**). These results show that ORP4 expression affected lipid droplet formation in the context of HCV replication. In addition, we examined the effect of CRLN and MβCD on lipid droplet formation in HCV RLuc cells. Similarly to ORP4 expression, both CRLN and MβCD treatments considerably increased lipid droplet formation in these cells (Figure **10B**). Furthermore, cells that were expressing ORP4 and treated with CRLN or MβCD appeared to produce more lipid droplets than each alone (data now shown). These results show that ORP4 expression alters intracellular lipid distribution. The apparent association between HCV inhibition and increased lipid droplet formation by ORP4 expression, CRLN and MβCD suggests that the lipid droplet content and distribution is important for HCV intracellular replication processes.

## Discussion

In the present study, we used the RNA affinity chromatography/2D/MASS strategy to isolate host proteins that are involved in HCV replication. The HCV 3’X RNA element in the 3' UTR has been shown to play a major role in HCV replication [4]. Thus, the 3’X RNA is potentially a center at which HCV proteins and host proteins form the replication complex for modulating HCV replication. Using the above-described strategy, host proteins were pulled-down from, identified, and compared among, cell lysates of HCV replicon-containing hepatocytes clone B, naïve hepatocytes Huh7.5 and non-hepatocytes 293T cells.

A total of 11 host proteins were identified to be in the 3’ X-containing replication complex by the combined RNA affinity chromatography/2D/MASS strategy (Table **1**). In addition to HMG-CoA synthase, the other host protein DDX25 could conceivably have a role in HCV replication. DDX25 belongs to the DEAD box protein family and is a putative RNA helicase characterized by a conserved DEAD motif. The functions of this protein family include energy-dependent unwinding of RNA-RNA and RNA-DNA duplexes, translation, nuclear and mitochondrial splicing, and ribosome and spliceosome assembly [51]. The DEAD box family is implicated in virus infection and replication: HCV core protein binds a DEAD box RNA helicase named DBX thereby inhibiting host cell mRNA translation [52]. Interaction between HCV core protein and another family member called DDX3 has been reported [53], and siRNA knockdown of DDX3 is known to impair HCV replication and virus production [54]. Moreover, another member of DEAD box protein family named p68 or DDX5 interacts with NS5B to inhibit HCV replication [55]. Thus, identification of HMG-CoA and DDX25 in the HCV replication complex supports the validity of the strategy.

Among the identified proteins is ORP4. Considering that cholesterol and lipid metabolism are closely linked to HCV replication and pathogenesis [31,44,56] and that the ORP family proteins play an important role in the regulation of cholesterol and lipid transport and metabolism [34-37], we decided to further investigate the role of ORP4 in HCV replication. ORP4 and other members of the ORP OSBP/ORP family associate with hVAP-A [40] (Figure **6**) and are implicated in sterol transport and/or signaling [34,36,37]. However, the function of ORP4 in HCV replication has not been investigated. We began by first determining ORP4 expression and intracellular localization in hepatocytes. Hepatocytes have a low level of constitutive ORP4 expression; but the overexpressed protein was localized in the ER (Figure **3**), which makes up the major intracellular 'membranous webs' for HCV replication [see a recent review [57]]. Importantly, ectopic expression of ORP4 decreased the luciferase reporter gene expression in the HCV subgenomic replicon cells (Figure **4A**) and the core expression in JFH-1-infected cells (Figure **4B**). Use of the naturally occurring ORP4S variant indicated that ORP4-mediated inhibition involved its sterol binding domain that is involved in transport (Figure **5**). Consistent with these findings, ORP4 expression did not increase cholesterol synthesis in hepatocytes and cholesterol production by these cells (Figure **9**); instead, it disrupted the lipid distribution dynamics, resulting in increased formation of lipid droplets in the context of HCV replication (Figure **10**).

HCV replication begins with assembly of the replication complex consisting of the viral RNA and nonstructural proteins and host proteins on the intracellular membrane system, mainly around the ER [58-64]. Compared to other identified proteins, the amount of ORP4 detected in the 3’ X-containing complex is the lowest (Figure **2**). The low expression level in hepatocytes and the liver [39] is the likely contributing factor. Lack of direct binding of ORP4 to 3’X RNA suggests that detection of ORP4 in this complex is mediated through unknown adaptor protein(s). We initially speculated that HCV RNA-dependent RNA polymerase NS5B is the candidate adaptor, as it binds to the ORP4-binding protein hVAP-A [40,43], which ORP4 also binds to [40]. Immunoprecipitation followed by Western blot analysis confirmed the presence of ORP4 in the NS5B-containing complex and/or the hVAP-A-containing complex (Figure **6**), which was consistent with the co-localization of ORP4 and NS5B in the ER (Figure **3**). The cell-based NS5B activity assay confirmed that ORP4 preferentially interfered with HCV NS5B polymerase activity (Figure **7**). ORP4 over-expression inhibited the reporter gene luciferase activity and HCV RNA synthesis in HCV RLuc subgenomic replicon cells (Figure **4A & B**). These results suggest that NS5B is the adaptor protein for ORP4 in the replication complex. ORP4 might inhibit NS5B and replication activity by the following mechanisms. First, ORP4 may affect the sub-cellular level and/or localization of viral nonstructural proteins whose localization on the ER membrane is critical for HCV replication. This possibility is supported by our finding that ORP4 interacted with NS5B (Figure **6**) and by previous studies which showed that ORP4 interacts with proteins and shuttle lipids between sub-cellular compartments [40,65-68]. Secondly, it is also possible that ORP4 acts through a host protein that is required for HCV replication. hVAP-A interacts with HCV nonstructural proteins (NS5A, NS5B) to mediate the formation of HCV RNA replication complex [41,42]. ORP4 interacts with hVAP-A and could contribute to the export of proteins from the ER, similar to OSBP [40]. Therefore, it is possible that by interacting with hVAP-A, ORP4 negatively affects the formation of the HCV replication complex and inhibits HCV replication. Third, it is also possible that ORP4 directly inhibits the enzymatic activity of HCV NS5B as demonstrated by the cell-based NS5B activity assay and thereby HCV replication. Fourth, a combined effect on both hVAP-A and NS5B could also contribute to the inhibition of HCV replication. We note that ORP4 interaction with a cellular protein does not exclude the possibility of it interacting with HCV nonstructural proteins. Whether one or more of these possible mechanisms characterize the effects of ORP4 on HCV RNA replication should be the subject of a follow-up study.

It has become increasingly evident that lipids are required for productive HCV replication. On one hand, HCV infection and replication up-regulate lipid synthesis and accumulation in the host [69-71]. On the other hand, replication complex formation and HCV replication takes place in the ER membranous web that is enriched in cholesterol and sphingolipid [72-74]. Drugs that affect the lipid biosynthetic pathway influence HCV replication [44,69]. For example, HCV RNA replication is inhibited by the HMG-CoA reductase inhibitor lovastatin, a potent inhibitor of cholesterol synthesis, and this inhibition is rescued by geranylgeranylation [31,33]. These findings suggest that HCV infection and replication likely give rise to a favorable intracellular lipid milieu that requires for productive HCV replication, and importantly point to a critical role of lipid molecules in HCV replication. However, our results that ORP4 expression had little effect on the lipid synthesis and production (Figure **9**) prompted us to examine the effect of ORP on intracellular lipid dynamics and distribution.

We next focused on lipid droplets. Lipid droplets are an organelle for the storage of neutral lipids such as triacylglycerol and cholesterol esters surrounded by a monolayer of phospholipids. They actively and dynamically move through the cytoplasm, interacting with other organelles such as the ER and participate in a range of functions including lipid homeostasis, cell signaling, and intracellular vesicular trafficking of lipids and proteins [see a review [75]]. We showed that ORP4 expression increased lipid droplet formation and that this only occurred in the presence of HCV (Figure **10**). HCV core protein expression has been linked to increased cytoplasmic lipid droplet formation in hepatocytes *in vitro* and *in vivo* [76-79], possibly by recruiting HCV nonstructural proteins and the replication complex to lipid droplets-associated membranes for assembly and production of infectious viruses around lipid droplets [80,81]. The inhibitory effect of ORP4 on HCV replication in the replicon system (Figures **4** & **5**) suggests that ORP4 likely regulates some intracellular lipid-related processes of HCV replication. Like other ORP protein, ORP4 could maintain the proper level and distribution of cholesterol by vesicle- or non-vesicle-mediated intracellular transportation of cholesterol [25,82-86]. Thus, it is possible that increased lipid droplet formation by ORP4 expression is the result of sequestration of “transportable” lipids and prevention of lipid transport-dependent assembly of the replication complex, resulting in inhibition of HCV replication. The other possibility is that increased lipid droplet formation by ORP4 expression disrupts the balance of lipid transport between the ER and other cytoplasmic organelles thus depleting the ER of lipids that are required for HCV replication. However, use of both the lipid synthesis inhibitor CRLN and the lipid sequester agent MβCD did not allow us to distinguish these two possibilities, as both inhibited HCV replication alone or in combination with ORP4 (Figure **8**), and both induced lipid droplet formation alone (Figure **10**) or in combination with ORP4 (data not shown). The unexpected finding that CRLN increased lipid droplet formation could be due to re-distribution of intracellular lipids in the absence of *de novo* lipid synthesis [87,88] or fusion of ER-derived lipid droplets [89]. Little change in lipid synthesis and production in ORP4-expressing cells (Figure **9**) supports the notion that ORP4-induced lipid droplet formation results from lipid re-distribution from the ER and not from new lipid synthesis. It is interesting to note that multiple attempts were made to knock down endogenous ORP4 in hepatocytes but all failed possibly as a result of negative effects on cell viability (personal communication by N. Ridgway). On the other hand, treatment of Huh7.5 cells with interferon-gamma did not lead any significant changes in endogenous ORP4 expression (data not shown). Thus, the low level of constitutive ORP4 expression in hepatocytes may have been selected to ensure its important function in host lipid metabolism but also provide a host control mechanism for HCV replication. Certainly, the exact underlying molecular mechanisms of ORP4-induced lipid droplet formation and inhibition of HCV replication merit further investigation.
